# Molecular Characterization of Poxviruses Associated with Tattoo Skin Lesions in UK Cetaceans

**DOI:** 10.1371/journal.pone.0071734

**Published:** 2013-08-13

**Authors:** Barbara A. Blacklaws, Anna M. Gajda, Sabine Tippelt, Paul D. Jepson, Rob Deaville, Marie-Francoise Van Bressem, Gareth P. Pearce

**Affiliations:** 1 Department of Veterinary Medicine, University of Cambridge, Cambridge, United Kingdom; 2 Faculty of Veterinary Medicine, Department of Pathology and Veterinary Diagnostics, Laboratory of Bee Diseases, Warsaw University of Life Sciences, Warsaw, Poland; 3 Department of Pharmacology, Toxicology and Pharmacy, University of Veterinary Medicine Hannover, Hannover, Germany; 4 Institute of Zoology, Zoological Society of London, London, United Kingdom; 5 Cetacean Conservation Medicine Group, Peruvian Centre for Cetacean Research (CEPEC), Berlin, Germany; University of Houston, United States of America

## Abstract

There is increasing concern for the well-being of cetacean populations around the UK. Tattoo skin disease (characterised by irregular, grey, black or yellowish, stippled cutaneous lesions) caused by poxvirus infection is a potential health indicatora potential health indicator for cetaceans. Limited sequence data indicates that cetacean poxviruses (CPVs) belong to an unassigned genus of the *Chordopoxvirinae.* To obtain further insight into the phylogenetic relationships between CPV and other *Chordopoxvirinae* members we partially characterized viral DNA originating from tattoo lesions collected in Delphinidae and Phocoenidae stranded along the UK coastline in 1998–2008. We also evaluated the presence of CPV in skin lesions other than tattoos to examine specificity and sensitivity of visual diagnosis. After DNA extraction, regions of the DNA polymerase and DNA topoisomerase I genes were amplified by PCR, sequenced and compared with other isolates. The presence of CPV DNA was demonstrated in tattoos from one striped dolphin (*Stenella coeruleoalba)*, eight harbour porpoises (*Phocoena phocoena)* and one short-beaked common dolphin (*Delphinus delphis*) and in one ‘dubious tattoo’ lesion detected in one other porpoise. Seventeen of the 18 PCR positive skin lesions had been visually identified as tattoos and one as a dubious tattoo. None of the other skin lesions were PCR positive. Thus, visual identification had a 94.4% sensitivity and 100% specificity. The DNA polymerase PCR was most effective in detecting CPV DNA. Limited sequence phylogeny grouped the UK samples within the odontocete poxviruses (CPV group 1) and indicated that two different poxvirus lineages infect the Phocoenidae and the Delphinidae. The phylogenetic tree had three major branches: one with the UK Phocoenidae viruses, one with the Delphinidae isolates and one for the mysticete poxvirus (CPV group 2). This implies a radiation of poxviruses according to the host suborder and the families within these suborders.

## Introduction

There is increasing concern for the well-being and viability of cetacean populations from the British Isles [1], [2]. Anthropogenic environmental modifications such as chemical and biological contamination are having detrimental effects on the health of these animals [1], [3]–[5] and their ecosystems [6]. Though the physical condition of cetaceans is difficult to monitor in the wild, there is mounting evidence that the presence, severity and epidemiology of some skin disorders may reflect population health [7]–[9]. Readily visually distinguished from other skin disorders, tattoo skin disease (TSD) is a potentially useful general health indicator a potentially useful general health indicatorfor cetaceans. In healthy populations, juveniles were reported to have a higher prevalence of TSD lesions than adults but in poor health short-beaked common dolphins (*Delphinus delphis*) and harbour porpoises (*Phocoena phocoena*) from the UK, adults showed a higher TSD prevalence than juveniles [8].

Cetacean poxviruses cause TSD characterised by irregular, grey, black or yellowish, stippled cutaneous lesions seen in several species of odontocetes and mysticetes [8], [10]–[12]. The family *Poxviridae,* subfamily *Chordopoxvirinae* infects a large number of vertebrates and currently comprises ten separate genera, viz: *Orthopoxvirus*, *Parapoxvirus*, *Capripoxvirus*, *Cervidpoxvirus*, *Crocodylidpoxvirus*, *Suipoxvirus*, *Leporipoxvirus*, *Yatapoxvirus*, *Avipoxvirus* and *Molluscipoxvirus* [13]–[16]. All *Poxviridae* replicate in the skin and mucosa to produce both localized (e.g. pseudocowpox and orf) and generalised lesions (e.g. smallpox and monkeypox) depending on the host-virus relationship [14], [15]. Limited sequencing of cetacean poxviruses suggested that they belong to an unassigned genus of the subfamily *Chordopoxvirinae* and include at least two groups: cetacean poxvirus (CPV)-1 in free-ranging and captive odontocetes from the US and Asia and CPV-2 in the mysticete *Balaena mysticetus* [17]. To obtain a further insight into the taxonomy and biology of the viruses affecting UK cetaceans, we characterized poxviruses originating from tattoo skin lesions collected in Delphinidae and Phocoenidae stranded along the coast of the UK between 1998–2008. We also evaluated the presence of CPV in skin lesions that were not recognized as tattoos in an attempt to determine the sensitivity and specificity of visual diagnosis for identifying TSD.

## Materials and Methods

### Sample Acquisition and Classification

Archived records of post-mortem examinations of UK cetaceans carried out between 1998 and 2008 at the Institute of Zoology, London, as part of the UK Cetacean Strandings Investigation Programme were searched for the mention of skin disorders. Post-mortem descriptions and digital photographs of the lesions were examined. They were classified as ‘tattoos’, ‘dubious tattoos’ or ‘other lesions' according to their gross characteristics [10], [12], [18]. Frozen samples of cutaneous lesions and healthy skin from 26 *P. phocoena*, 4 striped dolphins (*Stenella coeruleoalba*) and 1 *D. delphis* were processed for the PCR/molecular analysis (Table 1). Eight of them were also examined by transmission electron microscopy.

**Table 1:** PCR results of cetacean skin samples.

Animal code	Species	Sex	Age	Location ofstranding	Sample no.	Skin	18SrRNA*	β-globin*	DNApol#	DNAtopo#	PPV-DNApol#	PPV-up-do#	OPV-HA#
2002/262A	*P. phocoena*	F	J	Humberside	*8*	Normal	+++§	+++	–	–	–	–	–
2002/308	*P. phocoena*	M	A	Humberside	*9*	Normal	+++	+++	–	–	–	–	–
2003/296	*P. phocoena*	F	J	Humberside	*15*	Normal	+++	+++	–	–	–	–	–
2004/307A	*P. phocoena*	M	J	Humberside	*19*	Normal	+++	+++	–	–	–	–	–
2004/309A	*P. phocoena*	M	A	Humberside	*20*	Normal	+++	+++	–	–	–	–	–
2004/319	*P. phocoena*	M	A	Humberside	*21*	Normal	+++	+++	–	–	–	–	–
2007/85,1	*P. phocoena*	M	J	Humberside	*25*	Normal	+++	+++	–	–	–	–	–
2007/124	*P. phocoena*	M	A	Northumberland	*2*	Normal	+++	+++	–	–	–	–	–
1998/200	*S. coeruleoalba*	F	A	Avon	*6*	Normal	+++	+++	–	–	–	–	–
2002/178	*P. phocoena*	M	SA	Gwynedd	*29*	Lesion	ND	+++	–	–	ND	ND	ND
2002/382	*P. phocoena*	M	J	Norfolk	*30*	Lesion	ND	+++	–	–	ND	ND	ND
2004/342	*P. phocoena*	M	J	Pembrokeshire	*32*	Lesion	ND	+++	–	–	ND	ND	ND
2005/286	*P. phocoena*	F	J	Kent	*33*	Lesion	ND	+++	–	–	ND	ND	ND
2006/3	*P. phocoena*	F	J	Ceredigion	*34*	Lesion	ND	+++	–	–	ND	ND	ND
2006/41	*P. phocoena*	F	J	London	*35*	Lesion	ND	+++	–	–	ND	ND	ND
2006/228	*P. phocoena*	F	J	Kent	*36*	Lesion	ND	+++	–	–	ND	ND	ND
2007/85,2	*P. phocoena*	M	J	Humberside	*39*	Lesion	ND	+++	–	–	ND	ND	ND
2007/164	*P. phocoena*	M	SA	Gwynedd	*40*	Lesion	ND	+++	–	–	ND	ND	ND
2007/224	*P. phocoena*	M	SA	Carmarthenshire	*38*	Lesion	ND	+++	–	–	ND	ND	ND
2001/217	*S. coeruleoalba*	F	J	Devon	*28*	Lesion	ND	+++	–	–	ND	ND	ND
2003/215,2	*S. coeruleoalba*	F	A	Northumberland	*31*	Lesion	ND	+++	–	–	ND	ND	ND
2001/127	*P. phocoena*	M	A	Humberside	*7*	Lesion (? T)	+++	+++	++	+	–	–	–
2003/271	*P. phocoena*	F	A	Humberside	*12*	Lesion (old T)	+++	+++	++	+	–	–	–
					*13*	Lesion (old T)	+++	+++	++	+	–	–	–
					*14*	Lesion (old T)	+++	+++	++	++	–	–	–
2003/312	*P. phocoena*	F	A	Humberside	*16*	Lesion (T)	+++	+++	++	++	–	–	–
2004/267	*P. phocoena*	F	SA	Humberside	*17*	Lesion (T)	+++	+++	++	++	–	–	–
					*18*	Lesion (T)	+++	+++	++	+	–	–	–
2005/23	*P. phocoena*	F	A	Kent	*11*	Lesion (T)	+++	+++	++	+++	–	–	–
2005/28	*P. phocoena*	F	J	West Sussex	*22*	Lesion (T)	+++	+++	++	++	–	–	–
2006/223	*P. phocoena*	M	J	Devon	*23*	Lesion (old T)	+++	+++	++	+	–	–	–
					*24*	Lesion (old T)	+++	+++	++	+	–	–	–
2006/262	*P. phocoena*	F	SA	Dorset	*37*	Lesion (T)	ND	+++	++	++	–	–	–
2007/124	*P. phocoena*	M	A	Northumberland	*3*	Lesion (T)	+++	+++	+++	++	–	–	–
2000/4	*S. coeruleoalba*	F	A	Dorset	*1*	Lesion (T)	+++	+++	++	+	–	–	–
					*4*	Lesion (T)	+++	+++	+++	+	–	–	–
					*5*	Lesion (T)	+++	+++	+++	+	–	–	–
					*10*	Lesion (T)	+++	+++	++	–	–	–	–
2008/94.17	*D. delphis*	F	J	Cornwall	*41*	Lesion (T)	ND	+++	+	+	–	–	–
vaccinia	NA	NA	NA	NA	26	NA	+++	+++	++	++	–	–	+
sealpox	NA	NA	NA	NA	27	NA	+++	+++	–	+	+	+	–

Species: *P. phocoena -* harbour porpoise (*Phocoena phocoena*), *S. coeruleoalba* - striped dolphin (*Stenella coeruleoalba*), *D. delphis* – short-beaked common dolphin (*Delphinus delphis*); Sex: F - Female, M - Male; Age: A - Adult, SA - Sub-adult, J – Juvenile, estimated by length; (T) – tattoo lesion; (? T)- dubious tattoo lesion; (old T) - old tattoo lesion; PPV – parapoxvirus; OPV – orthopoxvirus;

* 10 ng DNA used as template;

# 50 ng DNA used as template;

§ number of+indicates relative amount of PCR product; ND - not done; NA – not applicable.

### Sample Processing and DNA Extraction

Skin samples were kept frozen whilst being processed. They were cut transversely through the skin and the blubber removed, to provide approximately 60–100 mg pieces for processing to genomic DNA. All samples were processed to extract total DNA using a DNAeasy Kit (Qiagen Ltd) according to the manufacturer’s instructions, with the difference that proteinase K digestion was performed overnight. DNA quality and content were evaluated by spectrophotometry using a ND1000 Nanodrop Spectrophotometer. The DNA was also tested using housekeeping gene PCR assays.

### Polymerase Chain Reaction (PCR)

PCRs contained 0.5 µM of each primer, 0.2 mM dNTP mixture, 1.5 mM MgCl_2_, 1× PCR buffer, 0.05% W-1, 2.5 U Taq DNA polymerase (Invitrogen Ltd), and water to a final volume of 15 µl for sample screening and 50 or 100 µl for further purification of PCR fragments. PCRs from skin samples using the OPV-HA and PPV-up-do primers produced smears which were reduced by the use of Platinum Hotstart Taq DNA polymerase (Invitrogen Ltd). Amounts of template used were 10 ng or 50 ng in screening PCRs and from 10 to 66.6 ng for PCRs used for purification of fragments. Water was used as the no template control, and as positive controls: vaccinia virus (vaccinia WR or MVA) DNA from infected cells (sample 26), and sealpox virus DNA from tissue (1/100 of stock, [19]) (sample 27) were used. PCR cycling conditions were as published for the majority of reactions but where there was poor recovery of product DNApol and DNAtopo assays were performed at annealing temperature 43°C with 2.5 mM MgCl_2_. The β-globin assay was performed as follows: 94°C for 2 min; then 40 cycles of 94°C, 30 s; 55°C, 30 s; 72°C, 60 s; and finally 72°C for 10 min.

Two housekeeping gene PCR assays were used to indicate the presence of genomic DNA in a sample and therefore suitability for use with more specific PCR assays. An assay for **18S rRNA** amplified an 186 bp DNA fragment [20] and an exon 2 specific **β-globin** assay amplified a 214 bp DNA fragment (designed by Dr Barbara Blacklaws using the Primer3 website; forward primer Bglobin e2 F1: CTGGTKGTCTACCCTTGGAC and reverse primer Bglobin e2 R1: AGTTCTCAGGATCCACRTGC). PCRs to detect conserved regions of all poxviruses were used, viz: the **DNApol** PCR assay used primers targeting the DNA polymerase gene (543 bp DNA fragment) [17]; the **DNAtopo** PCR used primers targeting the DNA topoisomerase I gene (344 bp DNA fragment) [17]. A PCR specific for the orthopoxvirus haemagglutinin gene was used to determine if samples were infected with orthopoxvirus, viz: the **OPV-HA** PCR (1138 bp fragment) [17]. Two PCRs specific for parapoxviruses were used, viz: the **PPV-up-do** assay used primers targeting the parapoxvirus major envelope B2L gene (84 bp DNA fragment) [20]; and the **PPV-DNApol** PCR used primers targeting the parapoxvirus DNA polymerase gene (536 bp fragment) [17].

### DNA Sequencing and Sequence Analysis

PCRs were repeated with samples chosen for the strongest bands and different animals (3, 4, 5, 7, 11, 14, 16, 17, 22, 24, 37 and 41) using the DNApol and DNAtopo assays. PCR products were purified using a PCR Purification Kit (Qiagen Ltd) then quantified by spectrophotometry on a Nanodrop Spectrophotometer. 20 ng of each purified PCR reaction was sequenced in both directions at the Department of Biochemistry, University of Cambridge. Sample 7 did not give readable direct sequencing results with either PCR product.

Sequences were analysed using EMBOSS [21] and ClustalW2 (http://www.ebi.ac.uk/Tools/clustalw2/). Phylogenetic trees were generated by maximum-likelihood probability with a PhyML model with 1000 bootstraps using nucleotide sequences that excluded the primer sequences and after gaps were determined from predicted amino acid comparison using SEAVIEW v4.2 [22] and trees drawn using Figtree v1.2.3 (Andrew Rambaut, Institute of Evolutionary Biology, University of Edinburgh; http://tree.bio.ed.ac.uk/). Sequence information for members of the *Chordopoxvirinae* was obtained from the GenBank repository through the NCBI website (http://www.ncbi.nlm.nih.gov/Genbank/): Swinepox AF410153; Lumpy skin disease AF325528; Goatpox NC_004003; Sheeppox NC_004002; Myxoma NC_001132; Rabbit fibroma NC_001266; Ectromelia AF012825.2; Rabbitpox AY484669.1; Cowpox DQ437593; Vaccinia AY243312.1; Horsepox DQ792504.1; Camelpox AF438165.1; Monkeypox AY603973.1; Variola DQ437592.1; Taterapox DQ437594.1; Volepox DQ066530; Muledeer NC_006966; Deerpox AY689437; Bovine papular stomatitis NC_005337; ORF NC_005336; Yaba monkey tumor NC_005179; Fowlpox NC_002188; Canarypox NC_005309; Nile crocodile poxvirus NC_008030; and Molluscum contagiosum NC_001731. DNA sequences generated from seal parapoxvirus isolates and cetacean poxvirus isolates (CPV) by Bracht et al. (2006) were also used. DNA polymerase sequences: steller sea lion parapoxvirus AY952942, AY952945, AY952948; spotted seal parapoxvirus AY780678; harbor seal parapoxvirus AY952939; CPV-1 AY952950, AY463004, AY463005, AY463006, AY463007, DQ071856, DQ071858, DQ071860, DQ071862; and CPV-2 AY846759. DNA topoisomerase sequences: steller sea lion parapoxvirus AY424954, AY952941, AY952944, AY952947; harbor seal parapoxvirus AY952938; CPV-1 AY952949, AY952951, DQ071857, DQ071859, DQ071861, DQ071863; and CPV-2 AY846760. Sequences derived here have been deposited with GenBank under Accession Numbers KC409036-KC409049 for DNApolymerase sequences and KC409050-KC409064 for DNA topoisomerase I sequences.

### Transmission Electron Microscopy

Frozen skin samples (4 PCR positive: 2000/4 [sample 4], 2003/271 [sample 14], 2003/312, 2005/23; 4 PCR negative: 1998/200, 2002/262A, 2002/308, 2007/85,1) were first fixed overnight at 4°C in 4% E-M Grade glutaraldehyde (Agar Scientific Ltd.) in 0.13 M phosphate buffer, washed then stored in 0.13 M phosphate buffer at 4°C until use. Samples were negatively stained in 2% osmium tetroxide (Oxkem Ltd, Oxon) in 0.13 M phosphate buffer overnight at room temperature. They were subsequently dehydrated through an alcohol gradient after which they were put in propylene oxide twice for 15 min each then transferred to equal parts of propylene oxide and resin (TAAB Embedding Resin) for 4 h at room temperature. Samples were placed in 2 changes of 100% resin over 24 h before placing in embedding resin mix (resin with Dodecenyl Succinic Anhydriade hardener, Methyl Nadic Anhydride and 2,4,6-Tris (Dimethylaminomethyl) phenol as accelerator, all components supplied by TAAB Laboratories Equipment Ltd.) and heated at 60°C for 24 h. Sections were visualised using a Hitachi Model H600 and photographed using Kodak Electron Microscope Film 4489.

## Results

### Samples and DNA

**Figure 1:**
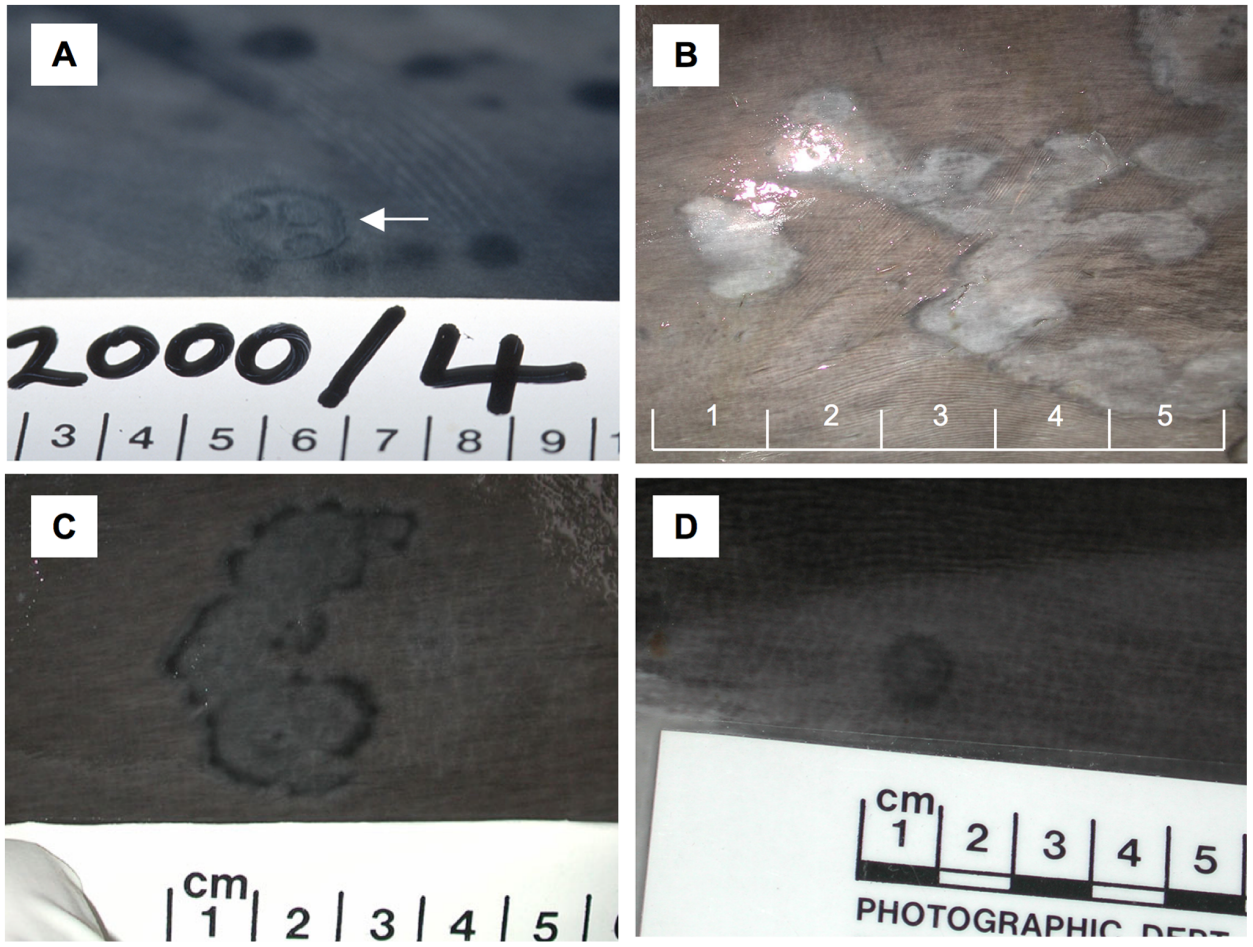
Images of cetacean tattoo skin disease. Tattoo lesions from **A**) *S. coeruleoalba* 2000/4; **B**) *P. phocoena* 2003/271; **C**) *P. phocoena,* 2003/312; and dubious tattoo lesion from **D**) *P. phocoena* 2001/127. The arrow in panel A indicates the tattoo lesion. The scale bars shown are in cm.

Typical tattoo skin lesions were recognized in 8 *P. phocoena*, 1 *S. coeruleoalba* and 1 *D. delphis* (Table 1, Figure 1). A lesion identified as a ‘dubious tattoo’ (a grey mark with a darker outline but without the typical stippled pattern) seen in another *P. phocoena* was also selected (Figure 1D). Uncharacterized skin lesions that did not match the characteristics of tattoos were collected in an additional 10 *P. phocoena* and 2 *S. coeruleoalba.* Healthy skin was sampled in seven TSD negative *P. phocoena,* one TSD positive *P. phocoena* (2007/124, sample 2) and a TSD negative *S. coeruleoalba.* All samples yielded DNA that could be used as a template in housekeeping gene PCR assays (18S rRNA and β-globin exon 2, Table 1).

### DNA Polymerase Gene Assay

**Figure 2:**
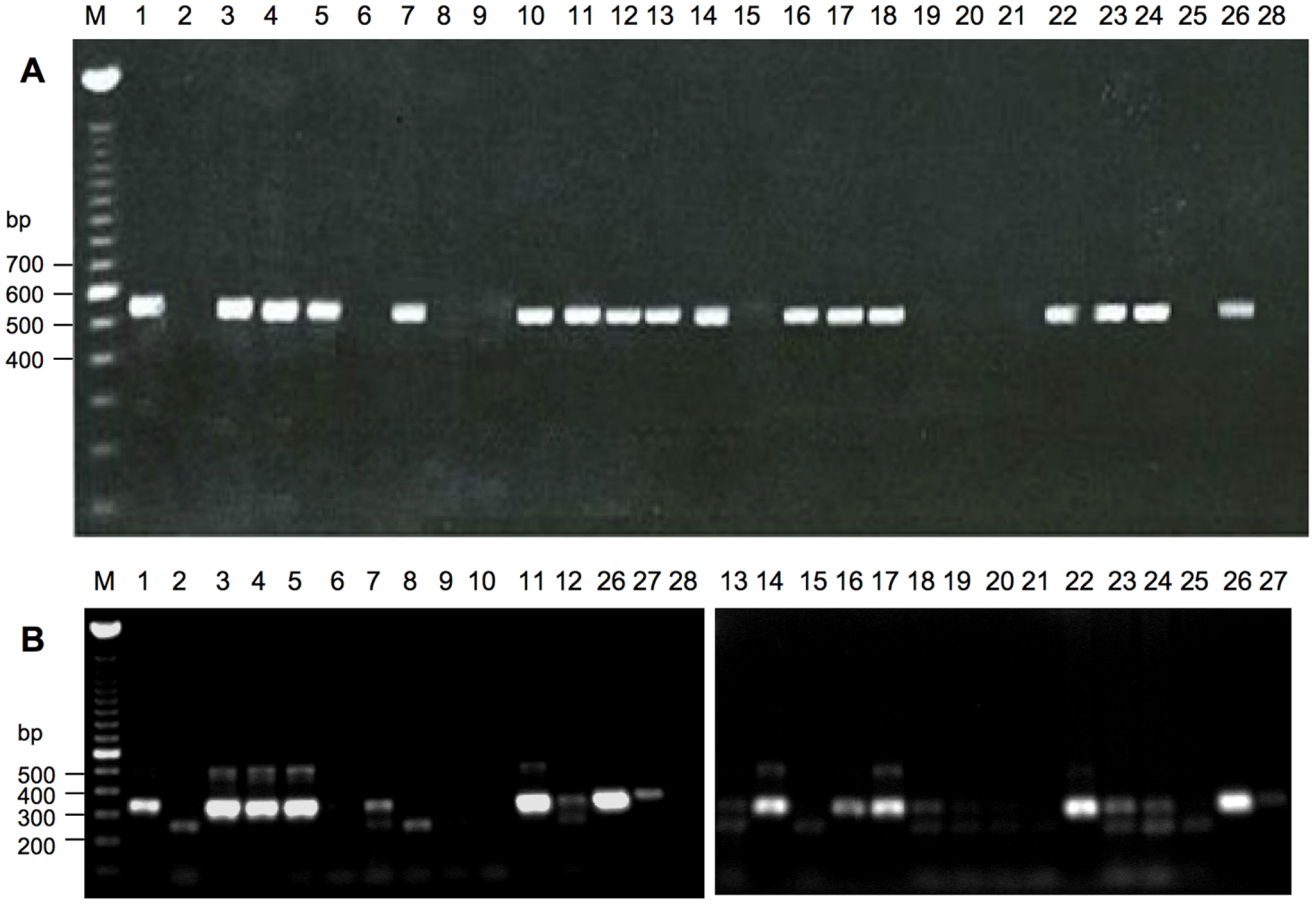
DNA polymerase and DNA topoisomerase I PCR assay results. Representative agarose gels of PCR products from reactions using 50 ng DNA. **A**) DNA polymerase assay (predicted product size 543 bp); **B**) DNA topoisomerase I assay (predicted product size 344 bp). M - 100 bp ladder; track numbers are for sample numbers; 1–25 skin samples; 26 vaccinia virus; 27 sealpox virus; 28 no template control.

Of the 30 cutaneous lesion samples from 23 individuals, 18 were positive using the DNA polymerase assay (Figure 2A and Table 1). All positive samples had been visually diagnosed as tattoos (N = 17) or dubious tattoo (N = 1). None of the healthy skin samples were positive. PCR positive samples from a *S. coeruleoalba* (samples 4 and 5), 9 *P. phocoena* (samples 3, 7, 11, 14, 16, 17, 22, 24, 37) and a *D. delphis* (sample 41) were chosen to perform larger PCR reactions for further purification and sequencing. Sample 7 did not give good sequencing results and so consensus sequence could not be analysed.

All sequences had G at position 16 of the forward primer (I in the primer). All products were 497 bp (excluding the primer sequences). Two groups were observed among the isolates originating from tattoo lesions sampled in the Phocoenidae, *P. phocoena*. Products from samples 3, 11 and 16 (group 1) were 100% identical as were samples 14, 17, 22, 24 and 37 (group 2). These two groups showed 99.8% nucleotide identity and 100% amino acid homology with each other. Among the Delphinidae, samples 4 and 5, collected from the same *S. coeruleoalba,* were identical and shared 95.8% identity at the nucleotide level and 100% similarity at the amino acid level with sample 41 originating from a *D. delphis.*

### DNA Topoisomerase I Gene Assay

An assay to detect DNA topoisomerase I DNA conserved across all the genera of the *Poxviridae* was also used. Results of this PCR were less clear than those obtained with the DNA polymerase test, with spurious sized products being seen and one of the lesion samples (no. 10) having no PCR product of the correct size although this was positive with the DNA polymerase PCR (Figure 2B and Table 1). This test also usually gave poorer DNA yields in comparison to its DNA polymerase counterpart. As with the DNA polymerase PCR product, the DNA topoisomerase I product from sample 7 did not give clean sequencing results and so consensus sequence was not analysed. Products were 302 bp long (excluding the primer sequences). Among the Phocoenidae, samples 3 and 17 (group 1topo) were 100% identical at the nucleotide level. Samples 11, 14, 16, 22 and 24 (group 2topo) were identical between themselves and only varied from group 1topo by 1 nucleotide. This did not cause a coding change. Sample 37 (*P. phocoena*) had 2 nucleotide differences with group 1topo and three changes compared with group 2topo that resulted in one amino acid difference.

Delphinidae samples 4 and 5 (from the same *S. coeruleoalba*) were identical to each other. These isolates had 97.4% homology at the nucleotide level and 100% homology at the amino acid level with isolate 41 (*D. delphis*). The *S. coeruleoalba* tattoo samples were only 88–89% identical at the nucleotide level and 91–92% identical at the amino acid level (8–10 amino acid substitutions) to the *P. phocoena* tattoo samples. These results indicate that the poxviruses infecting the Phocoenidae, *P. phocoena,* may belong to different species of cetacean poxviruses than those infecting the Delphinidae, *D. delphis* and *S. coeruleoalba*.

**Figure 3:**
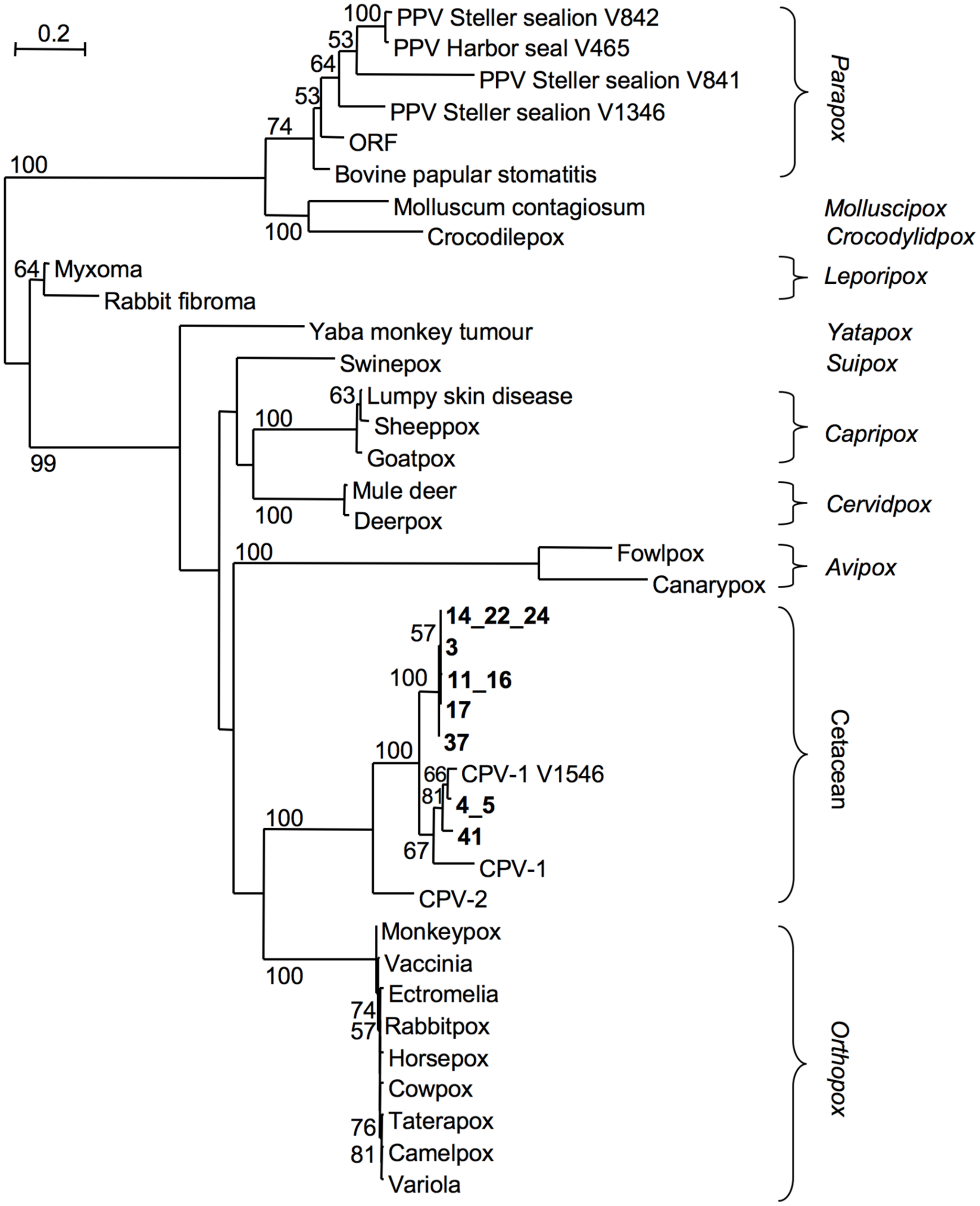
Phylogram of concatenated DNA polymerase and DNA topoisomerase I poxvirus sequences. Using published sequences and those from this study, the regions of DNA polymerase and DNA topoisomerase I amplified by PCR (without the primer sequences) were concatenated, aligned and a PhyML phylogram generated. Bootstrap values of branches have been shown if greater than 50. Cetacean samples with 100% nucleotide identity have been shown as one tip. The *Chordopoxvirinae* genera are shown with the proposed new cetacean genus.

The nucleotide sequences in the regions amplified (without the primer sequences) were concatenated and a phylogenetic tree drawn with other members of the *Chordopoxvirinae* (Figure 3). The results indicated that the UK dolphin and porpoise poxvirus isolates clustered with viruses of the CPV-1 group detected in captive Indo-Pacific bottlenose dolphins (*Tursiops aduncus*) and in free-ranging rough-toothed dolphins (*Steno bredanensis*), *S. coeruleoalba* and common bottlenose dolphins (*Tursiops truncatus*) from Florida [17]. The closest relationship of the UK isolates was with a sequence from a *T. truncatus* (GenBank accession numbers: DNA polymerase AY952950 and DNA topoisomerase I AY952950: CPV-1 V1546). In the DNA polymerase region sequenced, Delphinidae sample 4 shared 99.4% of identity and 100% amino acid similarity with this *Tursiops* isolate and in the DNA topoisomerase I region they shared 99% of identity and 100% amino acid similarity. Phocoenidae isolate 3 shared 98.2% identity and 99.4% amino acid similarity with *T. truncatus* isolate V1546 in the DNA polymerase region and in the DNA topoisomerase I region they shared 92% of identity and 98% amino acid similarity. The phylogenetic grouping shown in Figure 3 was similar when the DNA polymerase and DNA topoisomerase I fragments were analyzed separately (data not shown).

**Figure 4:**
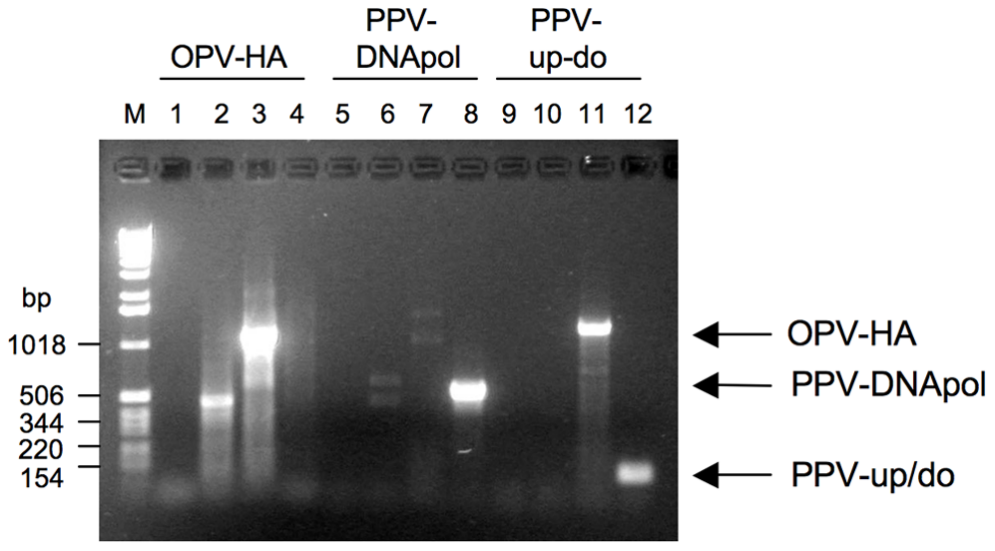
Orthopox and parapox virus specific PCR assay results. Representative agarose gel electrophoresis of products from PCR assays to detect orthopox or parapox viral DNA. Assays used 50 ng of template from one representative skin sample and appropriate controls. Tracks: M - 1 kbp ladder; 1, 5, 9 - no template control; 2, 6, 10 - sample 41; 3, 7, 11 - vaccinia virus; 4, 8, 12 - sealpox virus. PCR products from the OPV-HA assay (tracks 1–4, predicted size 1138 bp), the PPV-DNApol assay (tracks 5–8, predicted size 536 bp) and the PPV-up/do assay (tracks 9–12, predicted size 84 bp).

The PCR assays targeting the haemagglutinin gene of orthopoxviruses and the DNA polymerase and major envelope gene of parapoxviruses were negative on the tattoo lesions (Figure 4 and Table 1). This confirmed the phylogenetic tree grouping (Figure 3) that the cetacean samples sequenced here were neither *Orthopox* nor *Parapox* genera members [10], [11], [17].

### Diagnosis Specificity and Sensitivity

Results of the various PCRs run on the 39 frozen skin samples from two Delphinidae and one Phocoenidae species are shown in Table 1. All healthy skin samples and all 12 lesions classified as other than tattoos were negative for poxvirus DNA. Seventeen of the 18 skin lesions positive for poxviruses by PCR had been visually identified as tattoos and one as a dubious tattoo (Figure 1). Thus, visual identification of tattoo skin lesions had a 94.4% sensitivity and 100% specificity in comparison to the DNA polymerase PCR.

### Detection of Poxviruses by TEM

**Figure 5:**
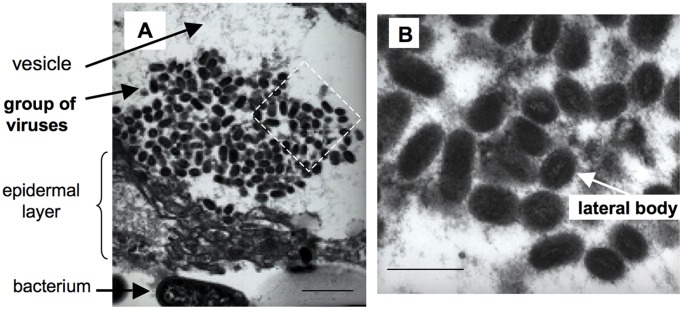
Transmission electron micrograph of a cetacean skin lesion. A transmission electron micrograph of sample 4 (*Stenella coeruleoalba* 2000/4) skin lesion. The box in micrograph **A**) has been shown at higher magnification in **B**). Magnification a) 12000×, bar = 1 um and b) 50 000×, bar = 0.4 um.

The presence of poxvirus in the tattoo lesions was also confirmed by TEM in a subset of the samples. Vesicles and cell disruption were observed in PCR positive tattoo skin samples (samples 4, 11, 14, and 16). Extracellular poxviruses grouped within vesicles under the skin surface were detected by TEM in the four tattoo samples (Figure 5). The virus particles were approximately 150 nm in diameter by 320 nm long and had the lateral bodies characteristic of *Poxviridae*. The cross-striations typical of parapoxvirus morphology were not seen on their surface.

## Discussion

The presence of viral DNA belonging to the recently described CPV-1 group was demonstrated in tattoo skin lesions from one *S. coeruleoalba*, nine *P. phocoena* and one *D. delphis* stranded along the shores of the UK between 1998–2008. It is the first time that CPV-1 has been identified in *D. delphis*, in cetaceans from European waters and in Phocoenidae worldwide.

Visual diagnosis of tattoo skin disease was confirmed to contain poxviruses by PCR and TEM results in all cases. Three kinds of morphology were observed: typical dark, irregular or rounded lesions with a stippled pattern representing the acute phase of the infection (Figure 1A, C), coalesced, circular, light grey blemishes with a darker outline corresponding to healing tattoos [18] (Figure 1B) and grey rounded marks surrounded by a darker ring (Figure 1D) [10]. The molecular and electron microscopic results demonstrate that visual detection of tattoos is 100% specific when conducted by experienced scientists.

The poxvirus DNA polymerase PCR assay used in this study was the most effective in identifying the presence of CPV DNA in the range of suspected lesions investigated. This PCR therefore offers considerable promise for routine use to enable definitive identification of the involvement of cetacean poxvirus in skin lesions observed in a variety of cetacean species around the world [8]. Where possible, PCR for the DNA polymerase gene should be used to confirm CPV infection in stranded dolphins, porpoises and whales carrying possible tattoo lesions.

The phylogenetic analysis carried out during the present study indicated some interesting relationships between cetacean poxviruses and other *Chordopoxvirinae* members, although the phylogenetic tree drawn here was from very small sections of two conserved genes and must be interpreted cautiously. Both the independent DNA polymerase and DNA topoisomerase I phylograms (not shown) and the concatenated sequence phylogram (Figure 3) indicated that the UK cetacean poxviruses were grouped most closely with those found in dolphins from the USA and Hong Kong to form a unique branch, separate from the known poxvirus genera and recently proposed as a new genus to be known as *Cetaceanpoxvirus* (CPV) [17]. From the sequence data available, CPV is only distantly related to the parapoxviruses infecting seals and sea lions. As CPV and parapoxviruses are the only poxviruses that have been isolated from cetaceans and pinnipeds respectively to date, it is unlikely that frequent cross-species infection occurs between these marine mammal groups.

The phylogenetic relationship revealed by the DNA comparisons confirm that CPVs are most closely related to members of the *Orthopoxvirus* genus [17]. There is also likely to be antigenic cross-reactivity between CPVs and orthopoxviruses as it has previously been shown that serum from Peruvian odontocetes is able to neutralise orthopoxvirus infectivity [23]. In the phylogenetic trees for the concatenated DNA polymerase and DNA topoisomerase I sequences, all the samples from UK *P. phocoena* grouped together in a lineage separate from the UK, US and HK Delphinidae isolates. This suggests that two different poxvirus lineages infect Phocoenidae and Delphinidae and that the CPV-1 group may contain several sub-groups specific for the different families of odontocetes. In the phylogenetic tree (Figure 3) there are three major cetacean branches, one with the UK *P. phocoena* viruses, one with the Delphinidae isolates and one for CPV-2, detected in the mysticete *B. mysticetus* [17]. This implies a radiation of poxviruses according to the suborder (odontocetes versus mysticetes) and the families within these suborders (Delphinidae and Phocoenidae). The geographic localization of CPV isolates may thus not produce as much genetic variation as expected but rather be influenced by the phylogenetic relationships of their host. Further studies should examine TSD samples collected in other odontocete families and in mysticetes worldwide to examine the validity of these conclusions. Taken together with the epidemiological characteristics in several odontocete species and populations [8], these data suggest that CPVs have co-evolved with cetaceans over many millennia and are likely very well adapted to their hosts.
